# Prediction value of the LACE index to identify older adults at high risk for all-cause mortality in South Korea: a nationwide population-based study

**DOI:** 10.1186/s12877-022-02848-4

**Published:** 2022-02-24

**Authors:** Eunbyul Cho, Sumi Lee, Woo Kyung Bae, Jae-ryun Lee, Hyejin Lee

**Affiliations:** 1 Department of Family Medicine, Seoul National University Bundang Hospital, 82 Gumi-ro, 173 Beon-gil, Bundang-gu, Seongnam-si, Gyeonggi-do 13620 Republic of Korea; 2 Health Promotion Center, Seoul National University Bundang Hospital, 82 Gumi-ro, 173 Beon-gil, Bundang-gu, Seongnam-si, Gyeonggi-do 13620 Republic of Korea

**Keywords:** LACE index, All-cause mortality, Nationwide database, Older adults

## Abstract

**Background:**

As a tool to predict early hospital readmission, little is known about the association between LACE index and all-cause mortality in older adults. We aimed to validate the LACE index to predict all-cause mortality in older adults and also analyzed the LACE index outcome of all-cause mortality depending on the disease and age of the participants.

**Methods:**

We used the National Health Insurance Service (NHIS) cohort, a nationwide claims database of Koreans. We enrolled 7491 patients who were hospitalized at least once between 2003 and 2004, aged ≥65 years as of the year of discharge, and subsequently followed-up until 2015. We estimated the LACE index using the NHI database. The Cox proportional hazards model was used to estimate the hazard ratio (HR) for all-cause mortality. Furthermore, we investigated all-cause mortality according to age and underlying disease when the LACE index was ≥10 and < 10, respectively.

**Results:**

In populations over 65 years of age, patients with LACE index ≥10 had significantly higher risks of all-cause mortality than in those with LACE index < 10. (HR, 1.44; 95% confidence interval, 1.35–1.54). For those patients aged 65–74 years, the HR of all-cause mortality was found to be higher in patients with LACE index≥10 than in those with LACE index < 10 in almost all the diseases except CRF and mental illnesses. And those patients aged ≥75 years, the HR of all- cause mortality was found to be higher in patients with LACE index ≥10 than in those with LACE index < 10 in the diseases of pneumonia and MACE.

**Conclusion:**

This is the first study to validate the predictive power of the LACE index to identify older adults at high risk for all-cause mortality using nationwide cohort data. Our findings have policy implications for selecting or managing patients who need post-discharge management.

**Supplementary Information:**

The online version contains supplementary material available at 10.1186/s12877-022-02848-4.

## Background

With the progression of the aging population, the medical expenses for older adults are also increasing [1]. This, along with the increase in human life expectancy has induced advances in medical research; however, it also presents many challenges to health and social care systems [2]. By 2050, there will be 1.6 billion older people worldwide, and South Korea, with the most rapidly aging population globally, will have the second largest aging population after Japan [3]. Thus, there is a concern that older and high-risk patient care will pressurize the health care finances and limited health resources. In the US, individuals in the top 5% of the total spending account for 35% of the total health spending [1]. Moreover, in many countries, high health care costs are concentrated in a small population [4–6]. In the US, 30% of the total Medicare budget is paid out on behalf of persons in their last year of life [7]. Caring for high-need and high-risk Americans with chronic disease or disabilities accounts for 84% of the US health care spending [8]. High-risk patients, such as terminally ill patients, patients with multiple chronic conditions or disabilities, and patients in the transition phase or in palliative care, are high-cost users of health care resources [9, 10]. Therefore, efforts to find and manage high-risk patients in advance and reduce readmissions and deaths for transitional patients who are discharged [11] on account of medical costs and quality are required.

The LACE index scoring tool, based on *length* of stay, *acuity* of admission, *comorbidities*, and *emergency* department visits, has been designed to predict early hospital readmissions and death [12] and has been widely used in many countries [13–15]. Although the LACE index is mainly used to predict readmission rates, our study additionally investigated the mortality rate for advance prediction of high-risk patients. The LACE index represents a cluster of features that indicate the health status of an individual; the higher the index score, the poorer their health and greater the risk of death [16]. Scores over a specific threshold can be used to “flag” at-risk patients for whom interventions may be appropriate [17]. The LACE index is simple and easy to calculate, and is routinely collected by all hospitals; it can thus be easily obtained from electronic health records by anyone.

Some risk scoring systems for predicting mortality after discharge are only applicable to specific patient populations and diseases [18–20]. Other models require the use of more lengthy formulas based on laboratory data and functional status or they are based on common geriatric syndromes [21–24]. However, it is difficult to generalize the conclusions to all institutionalized and community-dwelling individuals and can only be performed by experts [25]. Previous studies dealt with the performance of the LACE index for specific diseases [26–28] or institutions [29]. Therefore, this study aimed to validate the LACE index to predict all-cause mortality in South Korea’s older adults from a nationwide cohort. This study also analyzed the LACE index outcome of all-cause mortality depending on the disease and age of the participants.

## Methods

### Study design, participants, and setting

**Fig. 1:**
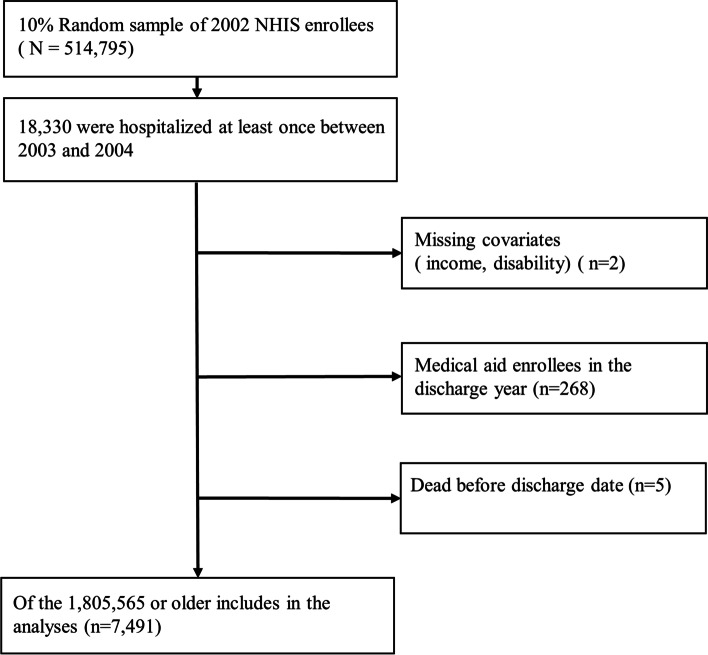
Flowchart of study participants

We used data from the NHIS-National Health Screening Cohort (NHIS-HEALS) from 2002 to 2015. The NHIS-HEALS cohort comprised a nationally representative random sample of 514,795 persons (10% random sample of all eligible) who were aged 40–79 years and had completed the NHIS health screening examination in 2002 or 2003 [30]. The NHIS is a mandatory universal public health insurance system that covers almost the entire Korean population, except medical beneficiaries in the lowest income bracket (~ 3% of the population) [31]. We analyzed prospectively collected data of consecutive admission episodes between January 1, 2003 and December 31, 2004. The NHIS collects information necessary for reimbursement for each medical service, including age, sex, disability status, monthly insurance premium (a proxy for household income), and disease codes. Patients’ information included primary diagnosis on admission, length of hospital stay, nature of admission, comorbidities, and number of emergency department visits. We studied a sample (*n* = 18,330) of NHIS enrollees who were hospitalized at least once between 2003 and 2004. If a patient had more than one hospitalization, the LACE index was calculated based on the first hospitalization. Of these, we analyzed only those aged ≥65 years as of the year of discharge. We excluded patients with missing covariates (income, disability), those with medical aid beneficiaries in the discharge year, those who dies before the discharge date, and patients with the same date of death and discharge, leaving 7491 patients for analysis (Fig. 1).

The primary outcome of the study was all-cause mortality according to the LACE index in older adults. We also analyzed all-cause mortality according to the LACE index to determine the effects of disease and age (65–74 and ≥ 75 years) in older adults.

All-cause death referred to all deaths, regardless of the cause. Comorbidities were identified using the Korean Standard Classification of Disease and Causes of Death-7 diagnosis, which is a modified version of the International Classification of Diseases, 10th revision (ICD-10). In this study, we chose high ranking diseases of cause of mortality in the elderly population and diseases of comorbidities of heavy burdens [32]. Pneumonia (J09–J18) and cancer (C00–C99) were defined based on the following ICD codes. Chronic renal failure (CRF) was defined as an estimated glomerular filtration rate < 60 mL/min/1.73 m^2^ (N18). Fractures were defined as follows: vertebral: S12.0, S12.1, S12.2, S22.0, S22.1, S32.0, M48.4, and M48.5; hip: S72.0, S72.1, and S72.2; upper arm: S42.0, S42.2, and S42.3; forearm: S52.5, and S52.6; and lower leg: S82.3, S82.5, and S82.6. Major adverse cardiovascular events (MACEs) were defined as composite outcomes of nonfatal stroke (I60, I61, I62, I63, I64), acute myocardial infarction (I21, I22, and I23), and death due to cardiac causes (I00–I99). Diabetes (DM) was defined as a history of related medications, or a fasting glucose level ≥ 126 mg/dL (E10–E15), and complications of DM were defined as E102–104, E112–114, E132–134, and E142–E144. We defined mental illness based on non-affective psychotic disorders (F20–24 and F28–29), affective psychotic disorders (F25, F30–31, F32.3, F33.3), anxiety-related and stress-related disorders (F40–48), alcohol or drug misuse (F10–16, F18–19), mood disorders without psychotic symptoms (F32–34, F38–39, excluding F32.3, F33.3), eating disorders (F50), and personality disorders (F60–63, 68–69).

The LACE index was calculated from the length of stay (scores 0–7), acuity of admission (score 0 or 3), comorbidity (scores 0–5), emergency department visits (score 0 or 4) with a scale ranging from 0 to 19, and the likelihood of outcome risk (mortality) with increasing scores [12]. Length of stay is defined as the duration of a single episode of hospitalization and the length of stay was extracted from the NHIS database. We identified comorbidities using International classification of Disease (ICD-10) codes in primary diagnosis, based on which we calculated the Charlson comorbidity index (CCI). (Supplementary Table 1) We compared patients with an index < 10 with those with an index ≥10 as previous studies have identified this as a useful criterion to differentiate high-risk patients [33].

### Statistical analysis

Baseline characteristics of individuals with LACE index < 10 were compared to those with LACE index ≥10 using a two-tailed Student’s *t-test* for continuous variables and χ^2^ test for categorical variables. Continuous variables are expressed as mean ± standard deviation (SD). A Cox proportional hazards regression model was used to estimate the associations between the LACE index and all-cause mortality. This study did not adjust for confounding variables because LACE index itself contains Charlson comorbidity index (CCI), it cannot be adjusted for comorbidities but we stratified the age. Results were presented as hazard ratio (HR) and 95% confidence interval (CI). Participants were followed up until death or the end of the study period (31 December 2015). The censoring date was the earliest of the following: date of death, or end date of the study period. We also obtained HR of all-cause mortality by the LACE index to estimate age-dependent effects. Statistical analyses were performed using SAS Enterprise Guide version 7.1 (SAS Institute, Cary, NC, USA) and R (version 3.3.3; The R Foundation for Statistical Computing, Vienna, Austria). Statistical significance was defined as a two-sided *p* value< 0.05.

The study design and protocol were reviewed and approved by the Institutional Review Board of the Seoul National University Bundang Hospital (IRB NO. X-2004-606-905). The requirement for informed consent was waived because the study was based on datasets that were otherwise open to the public. All methods were performed in accordance with the relevant guidelines and regulations, including the Declaration of Helsinki.

## Results

Of a total of 514,795 patients, 18,330 patients were hospitalized at least once between 2003 and 2004 (Fig. 1). Of them, 7491 patients aged ≥65 years were included in the study. The patient characteristics are described in Table 1.

**Table 1:** Characteristics of the patients over 65 years of age

Variables	Participants (*n* = 7491)	LACE score		*P*-value
		< 10	≥10	
		(*n* = 5746)	(*n* = 1745)	
Age(yr)				
Mean ± SD	70.9 ± 4.3	70.8 ± 4.4	70.9 ± 4.3	0.66
Distribution, n (%)				< 0.001
65–69	3396 (45.3)	2610 (45.4)	786 (45.0)	
70–79	3840 (51.3)	2931 (51.0)	909 (52.1)	
≥80	255 (3.4)	205 (3.6)	50 (2.9)	
Sex, n (%)				0.002
Male	4063 (54.2)	3059 (53.2)	1004 (57.5)	
Female	3428 (45.8)	2687 (46.8)	741 (42.5)	
Income, n (%)				0.13
1st quartile (low)	1488 (19.9)	1162 (20.2)	326 (18.7)	
2nd quartile	1058 (14.1)	792 (13.8)	266 (15.2)	
3rd quartile	1010 (13.5)	783 (13.6)	227 (13.0)	
4th quartile	1507 (20.1)	1130 (19.7)	377 (21.6)	
5th quartile (high)	2428 (32.4)	1879 (32.7)	549 (31.5)	
Disability, n (%)				0.05
Non-handicapped	7236 (96.6)	5564 (96.8)	1672 (95.8)	
Handicapped	255 (3.4)	182 (3.2)	73 (4.2)	
Pneumonia, n (%)				< 0.001
Yes	687 (9.2)	566 (9.9)	121 (6.9)	
No	6804 (90.8)	5180 (90.1)	1624 (93.1)	
Fracture, n (%)				0.004
Yes	921 (12.3)	742 (12.9)	179 (10.3)	
No	6570 (87.7)	5004 (87.1)	1566 (89.7)	
Cancer, n (%)				< 0.001
Yes	2479 (33.1)	1710 (29.8)	769 (44.1)	
No	5012 (66.9)	4036 (70.2)	976 (55.9)	
MACE, n (%)				
Yes	2849 (38.0)	2296 (40.0)	553 (31.7)	< 0.001
No	4642 (62.0)	3450 (60.0)	1192 (68.3)	
CRF, n (%)				< 0.001
Yes	135 (1.8)	83 (1.4)	52 (3.0)	
No	7356 (98.2)	5663 (98.6)	1693 (97.0)	
Diabetes, n (%)				
Yes	600 (8.0)	436 (7.6)	164 (9.4)	0.02
No	6891 (92.0)	5310 (92.4)	1581 (90.6)	
DM and complications of DM, n (%)				
Yes	646 (8.6)	477 (8.3)	169 (9.7)	0.08
No	6845 (91.4)	5269 (91.7)	1576 (90.3)	
Mental illnesses, n(%)				< 0.001
Yes	238 (3.2)	208 (3.6)	30 (1.7)	
No	7253 (96.8)	5538 (96.4)	1715 (98.3)	
LACE score				
Mean ± SD	7.5 ± 2.6			
Distribution, n (%)				
0–4	916 (12.2)			
5–9	4830 (64.5)			
≥10	1745 (23.3)			

The mean age of the study patients was 70.9 (SD = 4.3), and 54.2% of them were male. Approximately 38.0% of the patients had MACE and 33.1% had cancer. The mean LACE index was 7.5 (SD = 2.6), and among patients aged ≥65 years, 23.3% had a LACE index of ≥10. The proportions for those with LACE index ≥10 vs. LACE index < 10 in age group with 65–69 years were 76.9% vs. 23.1%, with 70–79 years 76.3% vs.23.7% and with ≥80 years 80.4% vs 19.6%.

Patients with a LACE index ≥10 tended to be male (*p* < 0.002); and have cancer (*p* < 0.001), CRF (p < 0.001), and Diabetes (*p* < 0.02). The baseline characteristics, except mean age, income, disability, and DM and complications of DM, were statistically significant between the patients with a LACE index < 10 and LACE index ≥10 (Table 1).

The hazard ratios (HRs) of all-cause mortality by LACE index in older adults are shown in Table 2. Among patients ≥65 years, those with a LACE index ≥10 had a significantly higher risk of all-cause mortality than those with a LACE index < 10 (HR: 1.44, 95% confidence interval [CI]: 1.35–1.54). In the analysis of all-cause mortality by each comorbidity in older adults, HR increased significantly when the LACE index was ≥10 in most diseases. When the LACE index ≥10, the HR was elevated in patients with pneumonia (1.63, 95% CI: 1.29–2.05), followed by MACE (1.61, 95% CI: 1.43–1.81), fracture (1.56, 95% CI: 1.25–1.95), diabetes (1.51, 95% CI: 1.20–1.89), and DM and complications of DM (1.50, 95% CI, 1.20–1.88). There were significant results in most diseases, but not in mental illness and CRF (Table 2).

**Table 2:** Hazard ratio of all-cause mortality by the LACE index in older adults

LACE score	Person-years	No. of events	Incidence	HR (95% CI)
Overall				
< 10	16,198,373	3155	54.9	Reference
≥10	4,072,806	1180	67.6	1.44 (1.35–1.54)
Pneumonia				
< 10	1,626,983	294	53.7	Reference
≥10	317,455	96	68.6	1.63 (1.29–2.05)
Fracture				
< 10	2,471,173	307	41.9	Reference
≥10	551,046	107	56.6	1.56 (1.25–1.95)
Cancer				
< 10	3,303,944	1194	70.9	Reference
≥10	1,422,545	602	75.8	1.14 (1.04–1.26)
MACE				
< 10	7,030,831	1124	49.5	Reference
≥10	1,465,509	385	66.4	1.61 (1.43–1.81)
CRF				
< 10	143,196	56	75.7	Reference
≥10	108,331	51	83.6	1.18 (0.80–1.72)
Diabetes				
< 10	1,348,802	225	52.4	Reference
≥10	447,469	112	65.5	1.51 (1.20–1.89)
DM and complications of DM				
< 10	1,489,295	243	51.7	Reference
≥10	467,580	114	64.8	1.50 (1.20–1.88)
Mental illnesses				
< 10	625,174	114	54.8	Reference
≥10	106,034	12	40.0	0.61 (0.34–1.11)

We subsequently evaluated the relationship between all-cause mortality in each age group according to comorbidities. In the 65–74 years age group, a LACE index ≥10 showed similar trends in the HRs for all-cause mortality, overall and for each disease (Table 3). In patients aged 65–74 years, the HR of all-cause mortality for a LACE index ≥10 for fracture, MACE, pneumonia were 1.74 (95% CI: 1.30–2.31), 1.72 (95% CI: 1.50–1.98), and 1.69 (95% CI: 1.27–2.26) respectively, whereas for those overall aged 65 years and older, the HR was pneumonia, MACE, fracture were 1.63(1.29–2.05), 1.61(1.43–1.81), 1.56(1.25–1.95) (Table 3). In patients aged 65–74 years, the HR of all-cause mortality for a LACE index ≥10 for diabetes, DM and complications of DM, and cancer was 1.66 (95% CI: 1.27–2.15), 1.65 (95% CI: 1.28–2.14), and 1.14 (95% CI: 1.02–1.27), respectively. In patients aged > 75 years, the HR of all-cause mortality for a LACE index ≥10 for pneumonia and MACE were 1.83 (95% CI: 1.23–2.70) and 1.36 (95% CI: 1.10–1.69), respectively. There were no significant outcomes in patients aged ≥75 years for fracture, cancer, CRF, diabetes, and DM and complications of DM. Moreover, mental illness was not significant regardless of age. For those patients aged 65–74 years, the HR of all-cause mortality was found to be higher in patients with LACE index≥10 than in those with LACE index < 10 in almost all the diseases except CRF and mental illnesses. And in patients aged 75 years and older, the HR of all-cause mortality was higher in patients with LACE index ≥10 than in patients with LACE index < 10 only in pneumonia and MACE (Table 3).

**Table 3:** Hazard ratio of mortality by the LACE index in older adults by age group

	all-cause mortality	
LACE score	65–74	≥75
Overall		
< 10	Reference	Reference
≥10	1.50 (1.39–1.63)	1.30 (1.15–1.48)
Pneumonia		
< 10	Reference	Reference
≥10	1.69 (1.27–2.26)	1.83 (1.23–2.70)
Fracture		
< 10	Reference	Reference
≥10	1.74 (1.30–2.31)	1.15 (0.81–1.62)
Cancer		
< 10	Reference	Reference
≥10	1.14 (1.02–1.27)	1.16 (0.95–1.43)
MACE		
< 10	Reference	Reference
≥10	1.72 (1.50–1.98)	1.36 (1.10–1.69)
CRF		
< 10	Reference	Reference
≥10	1.22 (0.77–1.92)	1.03 (0.52–2.05)
Diabetes		
< 10	Reference	Reference
≥10	1.66 (1.27–2.15)	1.21 (0.76–1.92)
DM and complications of DM		
< 10	Reference	Reference
≥10	1.65 (1.28–2.14)	1.20 (0.76–1.90)
Mental illnesses		
< 10	Reference	Reference
≥10	0.61 (0.30–1.27)	0.61 (0.22–1.71)

## Discussion

In this study, using nationwide cohort data, we found that hospitalized older patients those aged ≥65 years with high LACE index along with pneumonia, fracture, cancer, MACE, diabetes, and DM and complications of DM had a significantly higher risk of all-cause mortality when compared with patients with low LACE index. However, the HR of all-cause mortality was not significant in patients with mental illness and CRF and in those aged ≥75 years.

Since our study was the first to analyze the LACE index and mortality in a nationwide population, not limited to specific diseases, institutions, or hospitals, a direct comparison with previous studies was challenging. However, our results are consistent with those of a recent study of 2-year alive-discharge episodes from a single NHS hospital that reported an increasing trend in the risk of mortality in older patients with a LACE index ≥10 after hospital discharge [16]. In 2010, a Canadian study first proposed the LACE index to predict early death or unplanned readmission after discharge from the hospital to the community; however, it was limited to patients without cognitive impairment and excluded nursing home residents [12]. In our study, there were no significant outcomes for mental illness as opposed to the HR of all-cause mortality, which was significant for most diseases. As in the original study, there seems to be a limit to predicting mortality with the LACE index for mental diseases, including cognitive impairment. In a study that identified a potential predictor of 30-day psychiatric readmission, patients with severe medical comorbidities tended to be readmitted to nonbehavioral medical services and the burden of psychiatric comorbidities in patients with bipolar disorder did not affect readmission [34]. Although there is no direct study on the LACE index and mortality due to mental illness, the comorbidities accompanying mental illness seem act as risk factors rather than the mental illness itself; this could be because the number of comorbidities increase in patients with severe mental illnesses, such as bipolar disorder [34, 35]. Also in the Table 1, some comorbidities (pneumonia, fracture, MACE, and mental illnesses) showed a higher frequency in the LACE index < 10 group. Patients with chronic diseases such as cancer, CRF, and diabetes have long hospital stays and frequent emergency room visits [36]. On the other hand, acute diseases such as pneumonia, fracture, and MACE are more critical than chronic diseases, so there is a possibility that there could be many short-term deaths from diseases. Thus, the hospital stay is short, and emergency room visits are not repeated. Moreover, our study did not find significant results in CRF or mental illness in those aged ≥75 years, which could be because the number of patients was relatively small. In the case of elderly patients, study results show that cancer patients often die from unpredictable causes, such as death from cardiovascular disease or infection after hospitalization [37, 38]. Therefore, in patients aged ≥75 years, the HR for death in the LACE score 10 or higher group was not statistically significant in most comorbidity categories. However, there were significant results for life-threatening diseases, such as pneumonia and MACE, even in those aged ≥75 years; therefore, it is meaningful to predict mortality for those severe diseases with the LACE index even in small populations. Additionally, although the LACE index and mortality for MACE or pneumonia have not been previously studied, a higher LACE index in heart failure patients correlates with higher odds of 30-day readmission or death [39]. A study on the readmission rate of pneumonia patients showed that one in six patients were readmitted within 30 days after discharge [40]. Therefore, it seems that HRs and mortality among patients with high LACE index increases, as readmission rate of high risk patients with MACE or pneumonia increases.

In addition to the LACE index, prognostic indices, such as the Charlson comorbidity index (CCI), cumulative illness rating scale, geriatric index of comorbidity, and Kaplan, are also used. However, these are complex and require detailed information about patients and can only be performed by experts [25]. HOSPITAL (hemoglobin level at discharge, oncology at discharge, sodium level at discharge, procedure during hospitalization, index admission, number of hospital admissions, length of stay) scores are widely used to predict readmission; it has a fair discriminatory power but poor calibration in prediction readmissions [29]. However, the LACE index is meaningful in that it is simple and can be calculated using the claims database. Moreover, some studies showed that the LACE index showed better performance in predicting readmission, especially in certain diseases [17, 26, 27]. Furthermore, although there is no study directly comparing the CCI and LACE index, CCI is for cancer patients and the LACE index is for discharged patients; thus, LACE can be used preferentially for discharged patients to predict readmission and mortality.

There have been several validation studies on the readmission rate, but few studies have examined early death or mortality related to the LACE index. There have been only studies related to specific diseases such as neurosurgical diseases, [41] proximal humerus fractures, [27] and acute myocardial infarction [26]. Therefore, it is worthwhile to study not only the nationwide population but also patients depending on specific diseases for a long time, to derive a significant result between the LACE index and mortality. In addition, our study has the strength of not being biased because it was not conducted by a specific institution and it did not cover a selected population.

The geriatric population is heterogeneous and the majority of older adults have multiple morbidities; therefore, treatment approaches to older adults with specific diseases may be insufficient [2, 42] and identifying and predicting risk factors associated with early death is an important to achieve better quality of care. There have been numerous efforts and interventions to achieve this in the vulnerable elderly, such as the high-needs and high-cost patients (HNHC) [43] group, to improve overall patient outcomes and quality of life, and reduce healthcare spending. A proactive approach to addressing the problem of HNHC is to target interventions towards patients who are at risk of becoming HNHC [44]. This approach aimed at preventing at-risk patients from becoming HNHC involves identifying or predicting high-risk patients accurately before incurrence of substantial preventable or avoidable costs and deterioration of health status [45]. Several predictive models have been researched to reduce HNHC [44, 46]. Of them, the LACE index has been the most simple and easily predictive model to identify patients at risk of becoming HNHC healthcare users to date [12]. However, further research is required to predict additional factors that may increase the discrimination or accuracy of the index.

A limitation of our study is the possibility of inaccurate claims data. However, studies have shown that there is no problem in deriving such results as most of the claims data are accurate for many diseases [47]. Since our study used a retrospective design, the sample size or analysis method was not determined before data collection. To address this issue, we did not conduct artificial manipulation, such as cutting data or restricting the period. In this regard, there were some areas where significant results were not obtained due to the small number of people aged ≥75 years, and further research with a larger population is needed. Also this study did not adjust for confounding variables, such as age, sex, and income which may independently affect mortality in the general population. However the purpose of this study was to verify that it functions as an easily available tool in a real society not only in hospital, but also in government institutions or post-hospital transitional care services. Therefore this study have limitations in not adjusting for confounder, but it is meaningful that it took a phenomenological approach to see what value the LACE index has in society, based on cohort data targeting the entire nation. Finally, our study used the NHIS database; thus, information about the course of death or clinical implications of patients was inadequate. Therefore, further research on the causal relationship between patient mortality based on randomized controlled trials is required.

## Conclusions

This study revealed that the LACE index can be used to predict the risk of early death in older adults after discharge from the hospital, regardless of the disease, based on a large cohort using a national database. The LACE index could be an easy-to-use, validated tool to select or manage patients who need post-discharge management or those who need public intervention at the government level. Furthermore, our findings have important policy implications given the growing importance of cost containment and quality of care in healthcare systems, along with the increasing growth in the elderly population.

## Supplementary Information

**Additional file 1: Supplementary Table 1**. LACE index calculation.

## Data Availability

The data can be accessed on the National Health Insurance Data Sharing Service homepage of the NHIS (http://nhiss.nhis.or.kr). Applications to use the NHIS-HEALS data will be reviewed by the inquiry committee of research support and, once approved, raw data will be provided to the applicant with a fee. Although, the data are coded in English and numbers, use of individual data is allowed only for Korean researchers at the moment, but it would be possible for researchers outside the country to gain access to the data by conducting a joint study with Korean researchers.

## Abbreviations

NHIS: National Health Insurance Service
NHIS-HEALS: National Health Insurance Service-Health Screening Cohort
MACE: Major adverse cardiovascular event
DM: Diabetes mellitus
