# Supplementary material for: Prediction value of the LACE index to identify older adults at high risk for all-cause mortality in South Korea: a nationwide population-based study
Source: BMC Geriatr. 2022 Feb 24;22:154. doi: 10.1186/s12877-022-02848-4 (PMC8876396; doi:10.1186/s12877-022-02848-4)
Supplement: Supplementary file 1 — Additional file 1: Supplementary Table 1. LACE index calculation. [file 12877_2022_2848_MOESM1_ESM.docx]

**Supplementary Table 1. LACE index calculation**

| Attribute | Value | Score |
| --- | --- | --- |
| Length of stay | 1day | 1 |
|  | 2days | 2 |
|  | 3days | 3 |
|  | 4-6days | 4 |
|  | 7-13days | 5 |
|  | 14 or more days | 6 |
| Acute admission | Patient admitted to hospital via the emergency department. | 3 |
|  | Patient admitted to hospital via otherwise. | 0 |
| Comorbidities | Previous myocardial infarction | 1 |
|  | Cerebrovascular disease | 1 |
|  | Peripheral vascular disease | 1 |
|  | Diabetes without complications | 1 |
|  | Congestive heart failure | 2 |
|  | Diabetes with end organ damage | 2 |
|  | Chronic pulmonary disease | 2 |
|  | Mild liver or renal disease | 2 |
|  | Any tumor (including lymphoma or leukemia) | 2 |
|  | Dementia | 3 |
|  | Connective tissue disease | 3 |
|  | AIDS | 4 |
|  | Moderate or severe liver or renal disease | 4 |
|  | Metastatic solid tumor | 6 |
| Emergency visits in the 6 months prior to admission | 0 visit | 0 |
|  | 1 visit | 1 |
|  | 2 visits | 2 |
|  | 3 visits | 3 |
|  | 4 or more visits | 4 |
